# Plasma microRNA panel is a novel biomarker for focal segmental glomerulosclerosis and associated with podocyte apoptosis

**DOI:** 10.1038/s41419-018-0569-y

**Published:** 2018-05-10

**Authors:** Bin Xiao, Li-Na Wang, Wei Li, Li Gong, Ting Yu, Qian-Fei Zuo, Hong-Wen Zhao, Quan-Ming Zou

**Affiliations:** 1 National Engineering Research Center of Immunological Products, Department of Microbiology and Biochemical Pharmacy, College of Pharmacy, Third Military Medical University, Chongqing, 400038 China; 2 Department of Pharmacy, Southwest Hospital, Third Military Medical University, Chongqing, 400038 China; 3 Department of Kidney, Southwest Hospital, Third Military Medical University, Chongqing, 400038 China

## Abstract

Focal segmental glomerulosclerosis (FSGS) is a frequent glomerular disease, and is the common cause of nephrotic syndrome. However, there is no validated diagnostic blood biomarker for FSGS. Here, we performed a real-time PCR-based high-throughput miRNA profiling to identify the plasma signature for FSGS. We found four miRNAs (miR-17, miR-451, miR-106a, and miR-19b) were significantly downregulated in the plasma of FSGS patients (*n* = 97) compared with healthy controls (*n* = 124) in the training, validation, and blinded-test phases. The miRNA panel produced an AUC value of 0.82, and was associated with FSGS severity and histologic classification. A three-miRNA panel, including miR-17, miR-451, and miR-106a was related to FSGS remission. Furthermore, the downregulation of plasma-miRNA signature was not detected in disease controls (*n* = 119) such as IgA nephropathy (IgAN), mesangial proliferative glomerulonephritis (MSPGN), and membranous nephropathy (MN), and the miRNA panel discriminated between FSGS and disease controls. Pathway analysis showed that the four-miRNA panel may cooperatively regulate the pathways involved in the development of FSGS, such as apoptosis. We identified that phosphatase and tensin homolog (PTEN), Bcl-2-like protein 11 (BCL2L11), and chemokine (C-X-C motif) ligand 14 (CXCL14) were targets of miR-106a in human podocyte. Additionally, miR-106a overexpression suppressed podocyte apoptosis in vitro and the downregulation of four-miRNA panel probably resulted in the enhanced apoptosis in podocyte during FSGS development. Taken together, our data show that the plasma-miRNA panel is a potential independent diagnostic and prognostic factor for FSGS. Above miRNAs are involved in FSGS pathogenesis through regulating podocyte apoptosis.

## Introduction

Focal segmental glomerulosclerosis (FSGS) is a frequent glomerular disease characterized by proteinuria and podocyte injury. FSGS is the common cause of nephrotic syndrome, which accounts for about 40% of cases of the nephrotic syndrome in adults and 20% of cases in children^1,2^. Currently, the diagnosis of FSGS is based on biopsy results and there is no validated diagnostic blood biomarker for FSGS. Moreover, though there is some evidence showing clinical and pathologic features, such as proteinuria, the degree of renal insufficiency, the severity of fibrosis, etc., are associated with FSGS outcome, there is lack of plasma biomarkers to evaluate the prognosis and treatment response. Thus, there is a pressing need to identify diagnostic and prognostic noninvasive biomarkers for FSGS.

MicroRNAs (miRNAs) are a class of small noncoding RNA that negatively regulate gene expression by translational repression or induction of messenger RNA degradation^3^. Recently, miRNAs have been found to be implicated in the development of FSGS. For example, transgenic expression of miR-193a in mice induced podocyte effacement and FSGS^4^. In addition, miR-193a can function as a master switch to regulate the transdifferentiation of human parietal epithelial cells toward a podocyte phenotype^5^. MiR-30 could protect podocytes by targeting Notch1 and p53^6^. Moreover, miR-30 has been found to regulate calcium/calcineurin signaling in podocytes^7^. Increasing evidence suggests that circulating miRNAs can serve as novel, noninvasive biomarkers for various diseases, such as cancer, cardiovascular disease, organ transplant rejection, etc.^8–12^. Differently expressed circulating miRNAs have been found in patients with various forms of chronic kidney diseases (CKD) such as diabetic nephropathy, IgA nephropathy (IgAN), and lupus nephritis^13–15^.

In this study, we aimed to identify plasma-miRNA signature as a novel diagnostic and prognostic biomarker for FSGS, and tried to explore the role of miRNA panel in regulating the function of podocytes.

## Results

### Plasma-miRNA profiling and identification of miRNA signature in FSGS patients

**Fig. 1:**
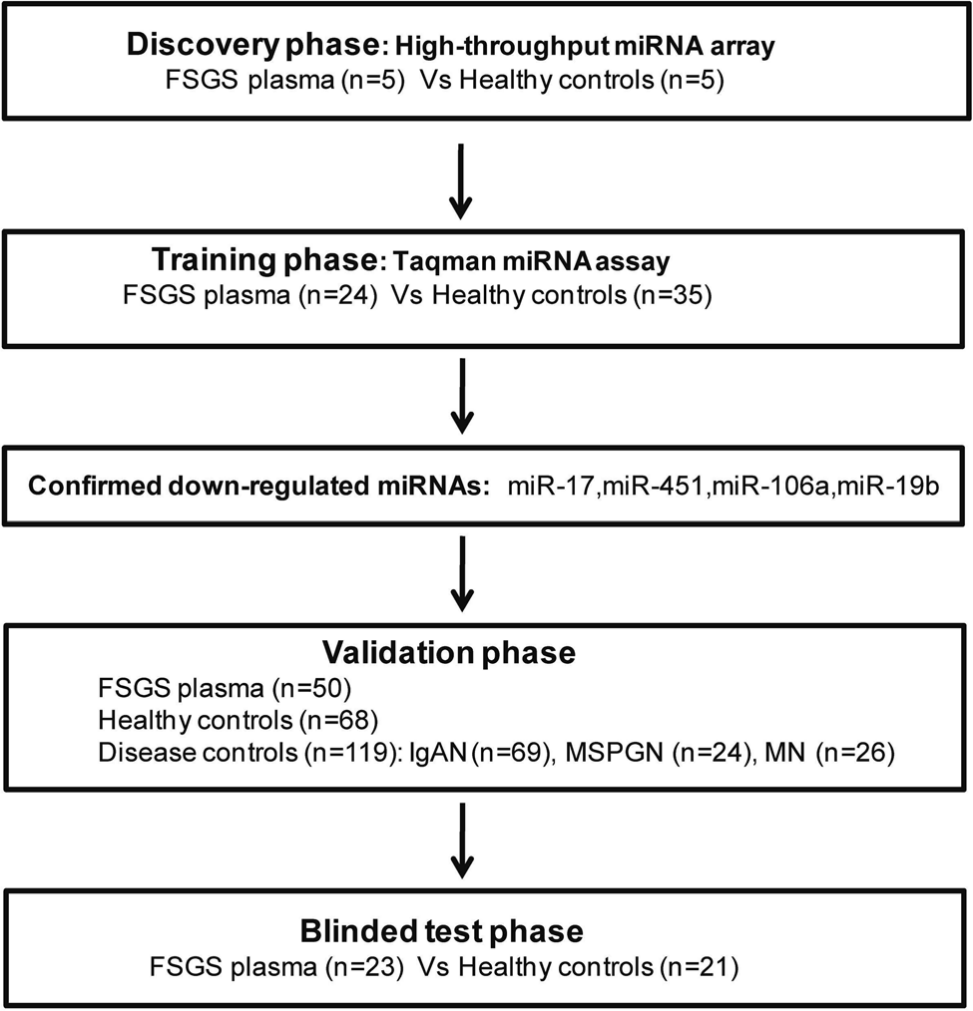
A diagram of sample collection and study design. A schematic of the study outlining the independent patients and samples used in discovery, training, validation, and blinded-test phases of the identification of plasma-miRNA panel for FSGS

**Fig. 2:**
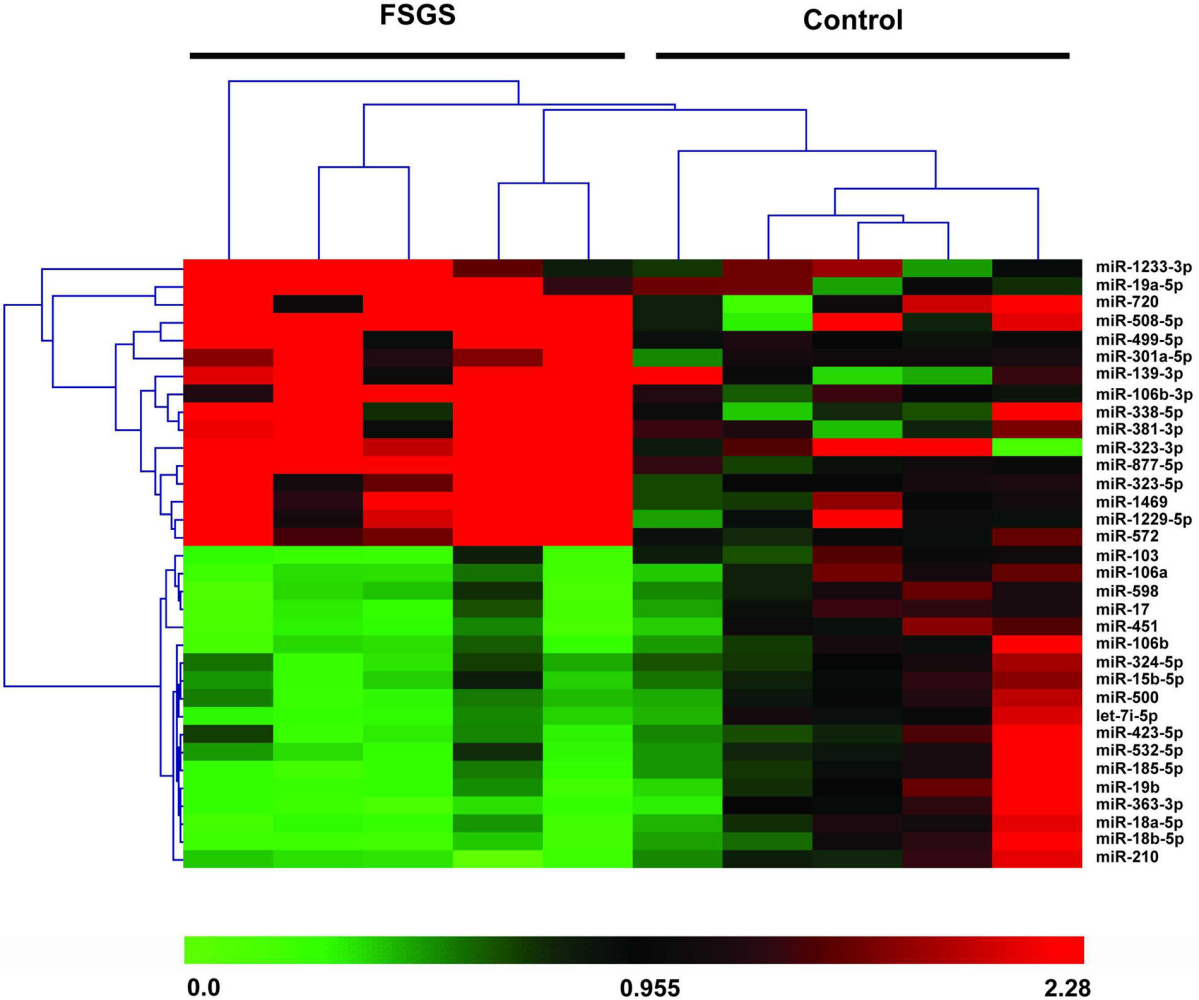
Hierarchical clustering analysis of differentially expressed miRNAs in plasma of FSGS patients in the screening phase. miRNA profiling in plasma from five FSGS patients and five healthy controls was performed by using a real-time PCR-based high-throughput miRNA array

To identify a diagnostic plasma-miRNA signature for FSGS, we performed a real-time PCR-based high-throughput miRNA profiling. A diagram of sample collection and study design is shown in Fig. 1. We identified a set of differentially expressed miRNAs in FSGS compared with healthy controls, including 16 upregulated and 18 downregulated miRNAs (*P* < 0.05, fold change >2) (Fig. 2). The delta Ct values of differentially expressed miRNAs were shown in Table S1. An ideal plasma-miRNA biomarker should be easily detected in FSGS patients or controls. According to this, we selected candidate miRNAs that satisfied two criteria for additional qRT-PCR (quantitative real-time polymerase chain reaction) validation: Cq values <28 in both FSGS and control groups; showing three-fold altered expression. As a result, four-plasma miRNAs (miR-17, miR-451, miR-106a, and miR-19b) were the ones which fulfilled above criteria and then selected for further validation.

**Fig. 3:**
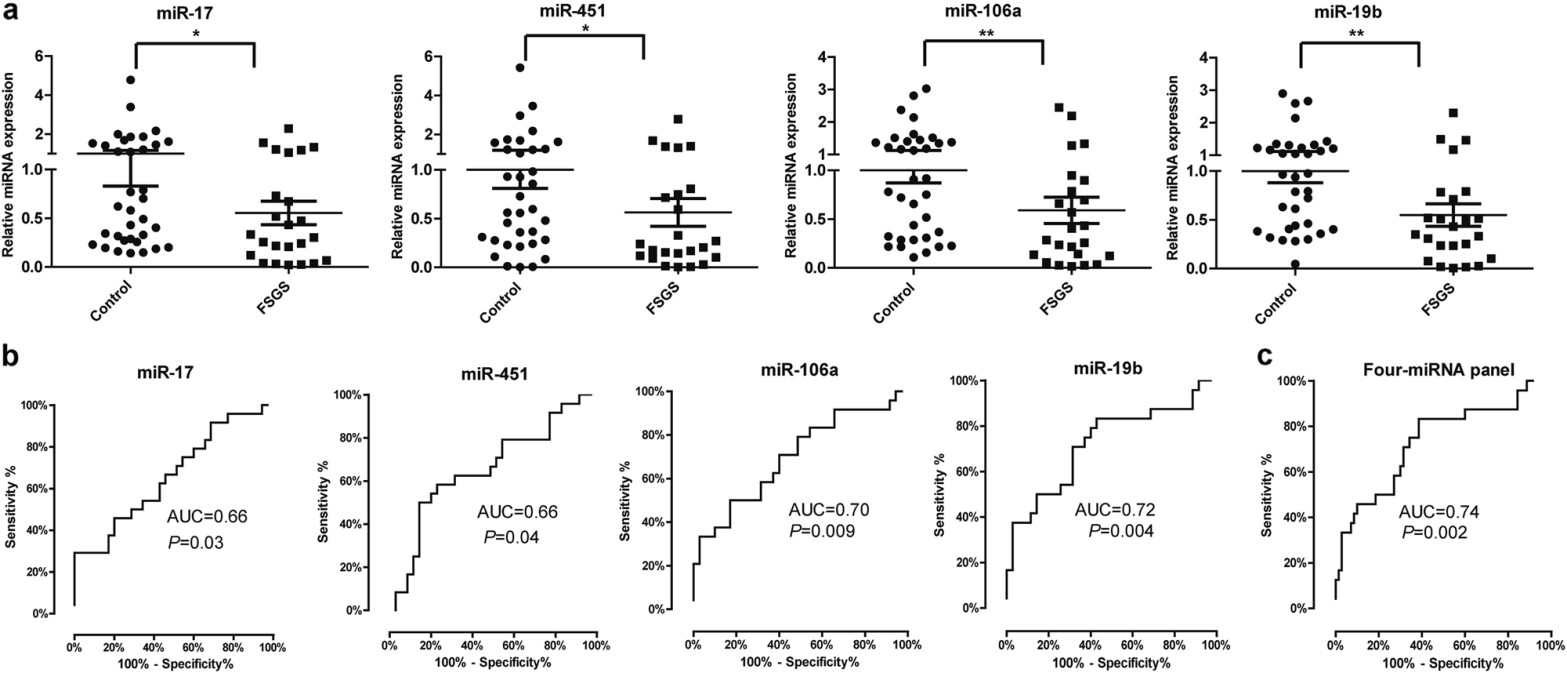
Expression of plasma-miRNA panel for FSGS diagnosis in the training phase. **a** Expression of miR-17, miR-451, miR-106a, and miR-19b in plasma of FSGS (*n* = 24) and healthy controls (*n* = 35). *P*-values were calculated using the Mann–Whitney test. **b** ROC analysis of individual miRNAs for the diagnosis of FSGS. **c** ROC analysis of four-miRNA panel for the diagnosis of FSGS. Logistic regression demonstrated that a linear combination of values for miR-17, miR-451, miR-106a, and miR-19b produced the best model for FSGS diagnosis. *P *<* 0.05, **P *<* 0.01

We then performed qPCR analysis of the four miRNAs in an independent training set consisting of 24 FSGS patients and 35 healthy controls. We found above plasma miRNAs were significantly downregulated in FSGS compared with healthy control. The fold changes in miR-17, miR-451, miR-106a, and miR-19b were 0.55, 0.56, 0.59, and 0.55, respectively (Fig. 3a).

To evaluate the sensitivity and specificity of the miRNAs in discriminating FSGS patients from healthy controls, we performed receiver operating characteristic (ROC) analysis. As shown in Fig. 3b, the areas under ROC curves (AUCs) of miR-17, miR-451, miR-106a, and miR-19b were 0.66 (95% confidence interval (CI), 0.52–0.80), 0.66 (95% CI, 0.51–0.80), 0.70 (95% CI, 0.56–0.84), and 0.72 (95% CI, 0.59–0.86), respectively. Next, we used logistic regression to determine whether the combination of four miRNAs exhibited better predictive value compared with individual miRNA. The AUC for the four-miRNA panel was 0.74 (95% CI, 0.60–0.87) (*P* = 0.002) (Fig. 3c). Taken together, our data suggest the four-miRNA panel may be a potential noninvasive biomarker for FSGS.

### Validation and blinded testing of the miRNA signature for FSGS

**Fig. 4:**
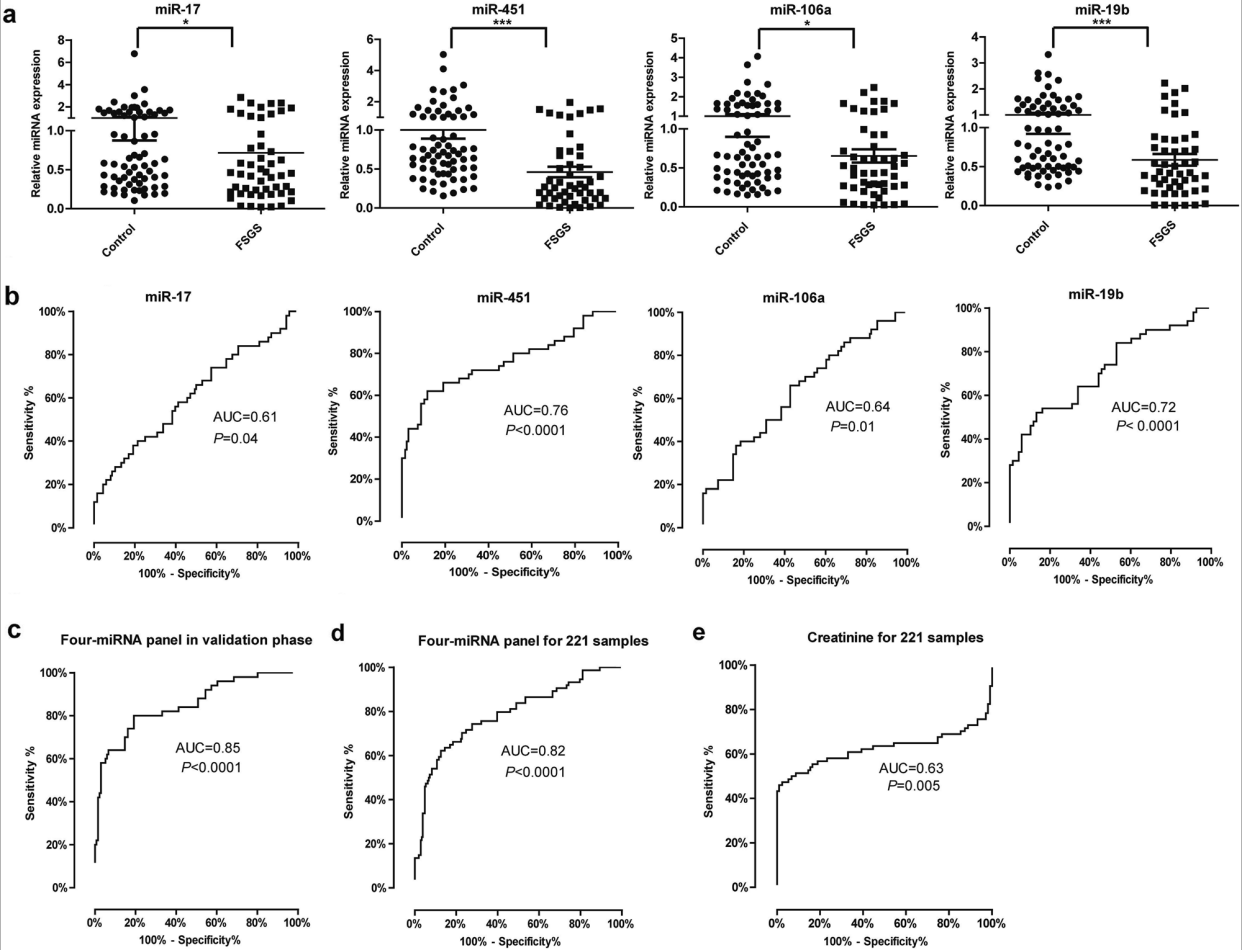
Expression of plasma-miRNA panel for FSGS diagnosis in the validation phase. **a** Expression of miR-17, miR-451, miR-106a, and miR-19b in plasma of FSGS (*n* = 50) and healthy controls (*n* = 68). **b** ROC analysis of individual miRNAs for the diagnosis of FSGS. **c** ROC analysis of four-miRNA panel for the diagnosis of FSGS in validation set. **d** ROC curves of the four-miRNA panel generated by analyzing all 221 samples from training, validation, and blinded-test sets. **e** ROC curve of serum creatinine for the diagnosis of FSGS in the training, validation and blinded-test phases. *P *<* 0.05, ***P *<* 0.001

We further analyzed the expression levels of the four miRNAs in an independent validation study consisting of 50 FSGS patients and 68 healthy controls. The consistent downregulation of the four miRNAs in plasma from FSGS patients was validated (Fig. 4a). ROC curve analysis showed that miR-17 had AUC of 0.61 (95% CI, 0.51–0.72), miR-451 had AUC of 0.76 (95% CI, 0.67–0.85), miR-106a had AUC of 0.64 (95% CI, 0.54–0.74), and miR-19b had AUC of 0.72 (95% CI, 0.62–0.82) (Fig. 4b). The AUC for four-miRNA panel was 0.85 (95% CI, 0.78–0.92) (*P* *<* 0.0001) with optimal sensitivity of 80.0% and specificity of 80.9% (Fig. 4c). In addition, we confirmed the downregulation of four miRNAs in FSGS with two different miRNA qPCR system (universal RT vs. stem-loop RT approach) (Figure S1).

We next verified the identified miRNA panel in a blinded study, including 23 FSGS patients and 21 healthy controls. We were blinded to the patients’ clinical characteristics when measuring miRNA levels. Based on the logistic regression model, we used the miRNA panel to discriminate FSGS or control. Our blinded-test results showed that the positive and negative predictive value of miRNA panel for FSGS was 87 and 81%. Finally, analysis of the data from training, validation, and blinded-test sets (*n* = 221) produced an AUC value of 0.82 (95% CI, 0.76–0.87) (*P* *<* 0.0001) (Fig. 4d). However, the AUC for serum creatinine was 0.63 (95% CI, 0.52–0.73) (Fig. 4e). Obviously, plasma-miRNA panel had better discriminating power in separating FSGS from healthy controls. Above data suggest that the four-miRNA panel can serve as a novel diagnostic biomarker for FSGS.

### Association of the miRNA biomarker panel with FSGS severity and histologic classification

**Fig. 5:**
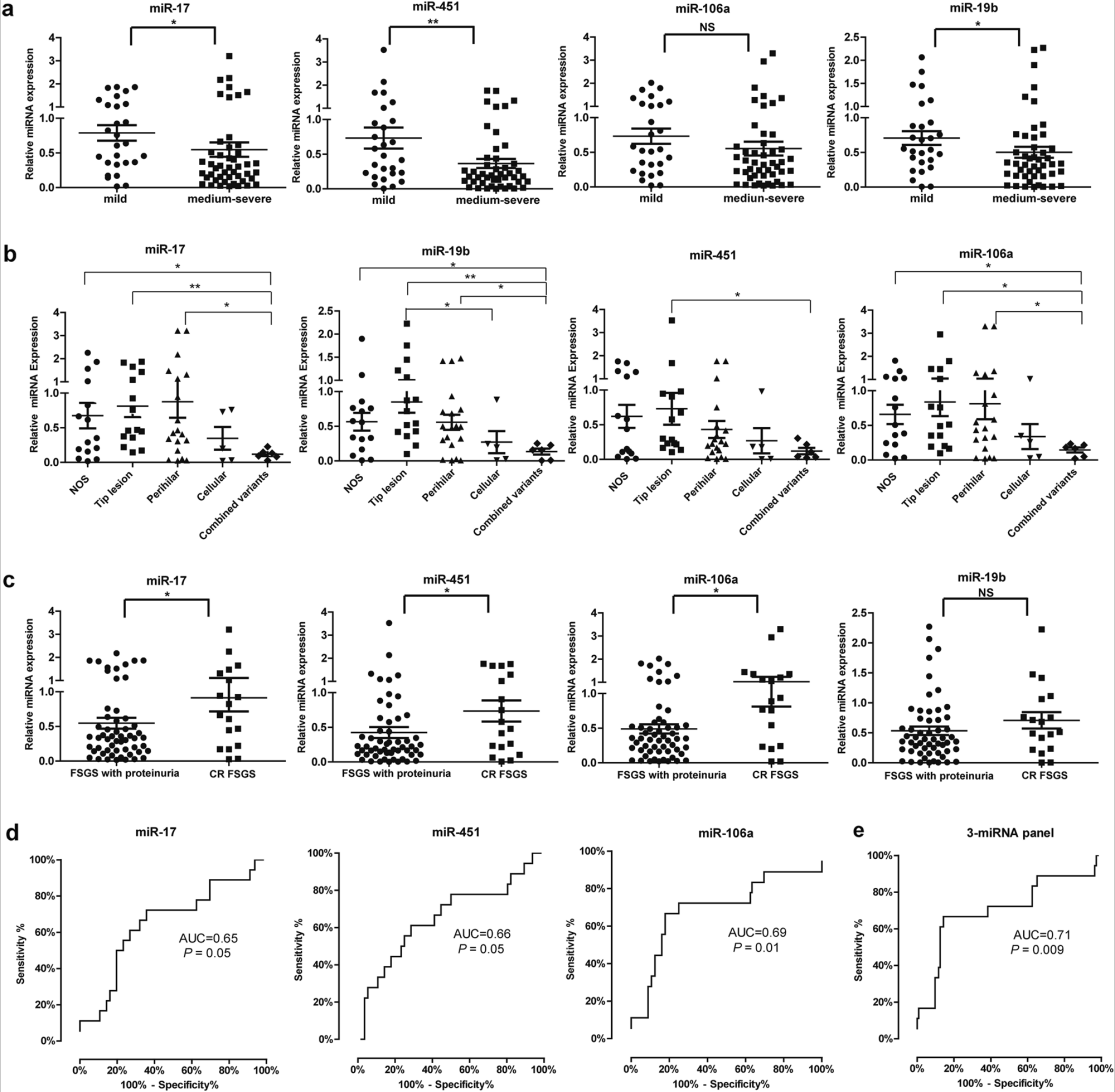
Association of the miRNA biomarker panel with FSGS severity and remission. **a** The expression of four-miRNA panel in FSGS patients with different grades of disease (CKD 1 (*n* = 27) vs. CKD 2–4 (*n* = 47)). **b** The expression of four-miRNA panel in FSGS patients with different subtypes of histologic classification. **c** The expression of four-miRNA panel between in FSGS patients with proteinuria (*n* = 56) and in FSGS patients with complete remission stage (urinary protein <400 mg/24 h after treatment) (*n* = 18). **d** ROC analysis of individual miRNAs for discriminating FSGS remission. **e** ROC analysis of three-miRNA panel including miR-17, miR-451, and miR-106a for discriminating FSGS remission. *P *<* 0.05, **P *<* 0.01

To determine whether the expression of miRNA panel was associated with FSGS progression, FSGS patients were divided into two groups according to CKD scores: mild FSGS (CKD 1) (*n* = 27) and medium-severe FSGS (CKD 2–4) (*n* = 47). We found that the expression of miR-17, miR-451, and miR-19b was significantly lower in medium-severe FSGS compared with mild FSGS (Fig. 5a).

Next, we compared the level of four miRNAs among different subtypes of histologic classification of FSGS. We categorized 61 FSGS samples with detailed histologic data into six histologic variants according to Columbia FSGS classification: glomerular tip lesion (*n* = 15), perihilar (*n* = 19), not otherwise specified (NOS) (*n* = 15), cellular (*n* = 5), collapsing (*n* = 1), and combined variants, which had more than one type of Columbia FSGS lesion (*n* = 6). We performed Kruskal–Wallis test, then found there was significant difference on the level of miR-17 (*P* = 0.04) and miR-19b (*P* = 0.01) between different subtypes of FSGS (Table S2). We further compared the levels of four miRNAs in each of the two subtypes. As shown in Fig. 5b, the plasma levels of miR-17, miR-19b, and miR-106a were significantly lower in combined variants group than in NOS, tip lesion, or perihilar groups. Above data suggest that there was a correlation between miRNAs expression (especially for miR-17 and miR-19b) and histologic classification of FSGS. However, larger scale of samples is needed to validate the conclusion.

### Plasma-miRNA panel is associated with FSGS remission

We then further examined whether plasma miRNAs could be associated with FSGS remission. Figure 5c showed miR-17, miR-451, and miR-106a were significantly downregulated in FSGS with proteinuria (*n* = 56) when compared with FSGS patients who were in remission (urinary protein <400 mg/24 h after treatment) (*n* = 18), whereas the expression of miR-19b did not differ in above two groups. ROC curve analysis showed that miR-17 had AUC of 0.65 (95% CI, 0.50–0.80), miR-451 had AUC of 0.66 (95% CI, 0.50–0.81), and miR-106a had AUC of 0.69 (95% CI, 0.53–0.85) (Fig. 5d). A three-miRNA panel, including miR-17, miR-451, and miR-106a had AUC of 0.71 (95% CI, 0.55–0.86) (*P* *<* 0.01) (Fig. 5e). Subsequently, we performed the Cox regression analysis to evaluate the association between the three-miRNA panel and FSGS remission. Univariate Cox analysis revealed that creatinine, serum albumin, estimated glomerular filtration rate (eGFR), CKD score, and three-miRNA panel were related to complete remission of FSGS. Furthermore, multivariate Cox regression analysis found that the expression of the three-miRNA panel was an independent related factor for complete remission of FSGS (OR = 2.868, 95% CI: 1.090–7.545, *P* *=* 0.033), after adjusting for creatinine, eGFR, CKD score, serum albumin, and treatment type (Table S3). Above data suggest that plasma-miRNA panel is probably associated with FSGS remission.

### Disease specificity of downregulation of four-miRNA panel

**Fig. 6:**
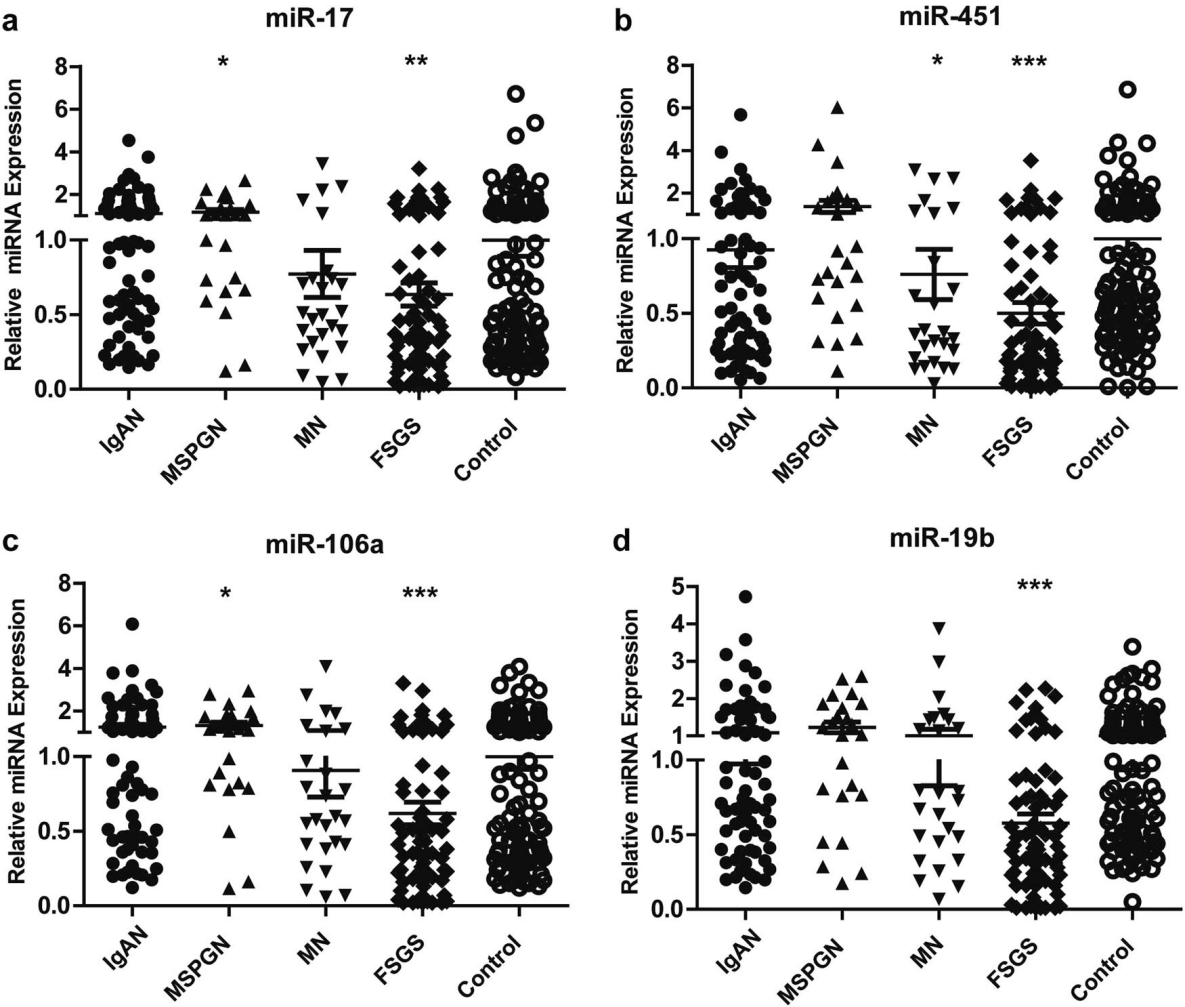
Disease specificity of downregulation of four-miRNA panel in FSGS. a-d The expression of miR-17, miR-451, miR-106a, and miR-19b between in FSGS patients (*n* = 74) and in other chronic kidney diseases including 69 IgAN patients, 24 MSPGN patients, and 26 MN patients. *P *<* 0.05, **P *<* 0.01, ***P *<* 0.001

To determine whether the downregulated expression of four-miRNA panel was specific to FSGS or associated with other CKD, we enrolled 69 IgAN patients, 24 mesangial proliferative glomerulonephritis (MSPGN) patients, and 26 membranous nephropathy (MN) patients as disease controls. qRT-PCR results showed that the levels of miR-17, miR-451, miR-106a, and miR-19b were the lowest in FSGS patients compared with healthy controls and disease controls. We did not observe significant downregulation of four-miRNA panel in plasma samples from disease controls compared with health controls (Fig. 6). We then analyze the ability of plasma miRNAs biomarkers to distinguish between the FSGS and disease control. As shown in Figure S2, plasma levels of above four miRNAs was able to significantly discriminate between FSGS and disease controls (IgAN, MSPGN) (AUC: 0.63–0.79).

Above data suggest that the downregulation of four-miRNA panel is specific to FSGS and above miRNAs may be involved in the pathogenesis and progression of FSGS.

### Expression correlation within the four-miRNA panel and correlation analysis of miRNAs and clinical parameters

We performed correlation analysis to test whether the expression of the four miRNAs were correlated with each other. Correlation analysis results showed that the levels of the four miRNAs in the panel were significantly positive correlated with each other (Table S4). For example, significant positive correlations were found between miR-17 and miR-451, and between miR-106a and miR-19b plasma levels, with correlation coefficient values of 0.773 and 0.843, respectively (*P* *<* 0.001) (Figure S3). To exclude the influence of correlation within four miRNAs on the model, we did colinearity diagnostics analysis by SPSS. The results showed there was no significant multicollinearity among the four miRNAs, because VIF values <10 (Figure S4).

We next examined the relationship between four-miRNA panel and clinical parameters, including creatinine, eGFR, proteinuria, serum albumin, and treatment type. However, no correlation was found (Table S5). Above data suggest that plasma-miRNA panel is an independent biomarker for FSGS patients.

### Identification of target genes of four-miRNA panel and pathway analysis

To further investigate the function of four-miRNA panel in the development of FSGS, we predicted target genes, which can be co-regulated by four-miRNA panel by using the prediction algorithms, MICRORNA.ORG. We found a list of 544 potential targets and David pathway analysis showed that the top five common pathways targeted by four-miRNA panel are involved in pathways in cancer, PI3K-Akt signaling pathway, FoxO signaling pathway, focal adhesion, and calcium signaling pathway, etc. (Table S6). Then we chose PTEN, BCL2L11, Caspase-7 (CASP7), C-X-C motif CXCL14, and collagen type I alpha 2 (COL1A2) as the candidate targets because they are closely related with the process of glomerular injury such as apoptosis, fibrosis, and scarring.

**Fig. 7:**
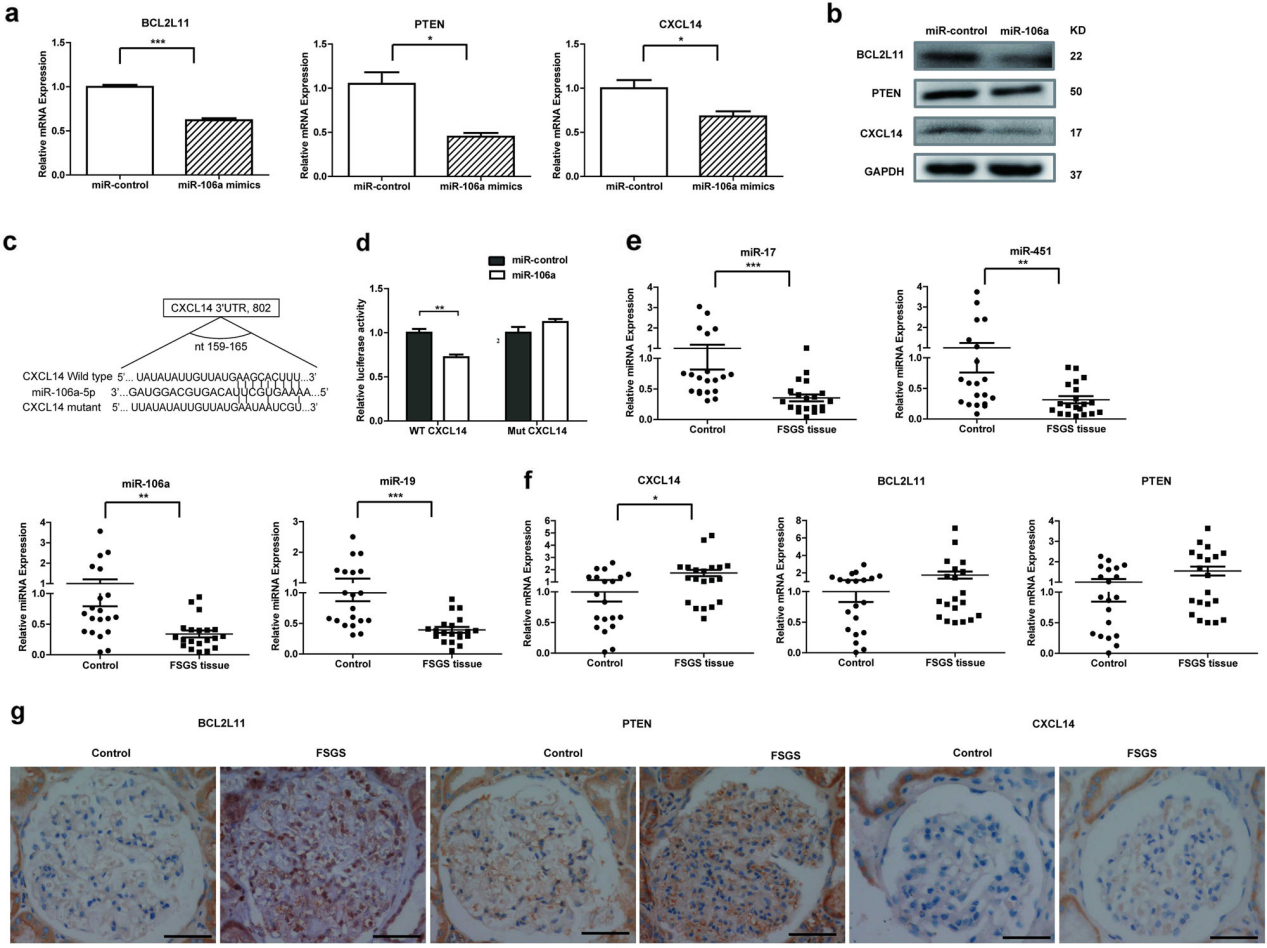
Prediction of pathways co-regulated by four-miRNA panel and identification of targets of miR-106a in human podocyte during FSGS development. **a** The relative mRNA expression of target genes in miR-106a or miR-control transfected human podocytes. **b** The protein expression analysis of target genes by western blotting in miR-106a or miR-control transfected human podocytes. **c** Wild-type or mutant binding sites of CXCL14 3′-UTR for miR-106a. **d** HEK293 cells were transiently co-transfected with luciferase report vectors containing wild-type or mutant CXCL14 3′-UTR, and either miR-106a mimics or miR-control. Luciferase activities were normalized to the activity of Renilla luciferase. **e**, **f** The relative expression of four-miRNA panel and target genes in 20 set of FFPE FSGS tissue specimens and FFPE normal renal tissues. **g** Immunohistological staining of BCL2L11, PTEN, and CXCL14 in FSGS renal biopsies and normal renal tissues. Scale bars = 20 μm. *P *<* 0.05, **P *<* 0.01, ***P *<* 0.001

Among the four miRNAs, we selected miR-106a as a representative for identification of targets. We found overexpression of miR-106a in podocytes resulted in the downregulation of mRNA and protein levels of PTEN, BCL2L11, and CXCL14 (Fig. 7a, b), while miR-106a had no effect on the expression of CASP7 and COL1A2 in podocytes. Since PTEN has been reported to be potent target of miR-106a^16^, and miRTarBase database provided experimental evidences to support miR-106a:BCL2L11 interaction (detailed information is showed in the following link: http://mirtarbase.mbc.nctu.edu.tw/php/detail.php?mirtid=MIRT494539#evidence), so we only focused on CXCL14 and performed luciferase assays (Fig. 7c). The relative luciferase activity was reduced by miR-106a in the vector containing the CXCL14 3′-UTR. However, miR-106a had no effect on mutant vectors (Fig. 7d). To confirm the relation between four-miRNA panel and targets expression, we examined the expression of above four miRNAs and target genes (PTEN, BCL2L11, and CXCL14) in 20 set of formalin-fixed, paraffin-embedded (FFPE) FSGS tissue specimens and FFPE normal renal tissues. Consistent with plasma miRNAs expression, above miRNAs were significantly downregulated in FSGS tissues (Fig. 7e). As shown in Fig. 7f, CXCL14 was significantly upregulated in FSGS tissues compared with normal renal tissues. The expression of PTEN and BCL2L11 showed increased trend in FSGS tissues, though it did not reach statistical significance. In addition, immunohistochemistry results showed that higher protein levels of PTEN, BCL2L11, and CXCL14 were observed in FSGS renal biopsies compared with normal renal tissues (Fig. 7g). Taken together, above data suggest that enhanced expression of PTEN, BCL2L11, and CXCL14 in FSGS could be a result of reduced four-miRNA panel expression.

### MiR-106a suppresses podocyte apoptosis in vitro

**Fig. 8:**
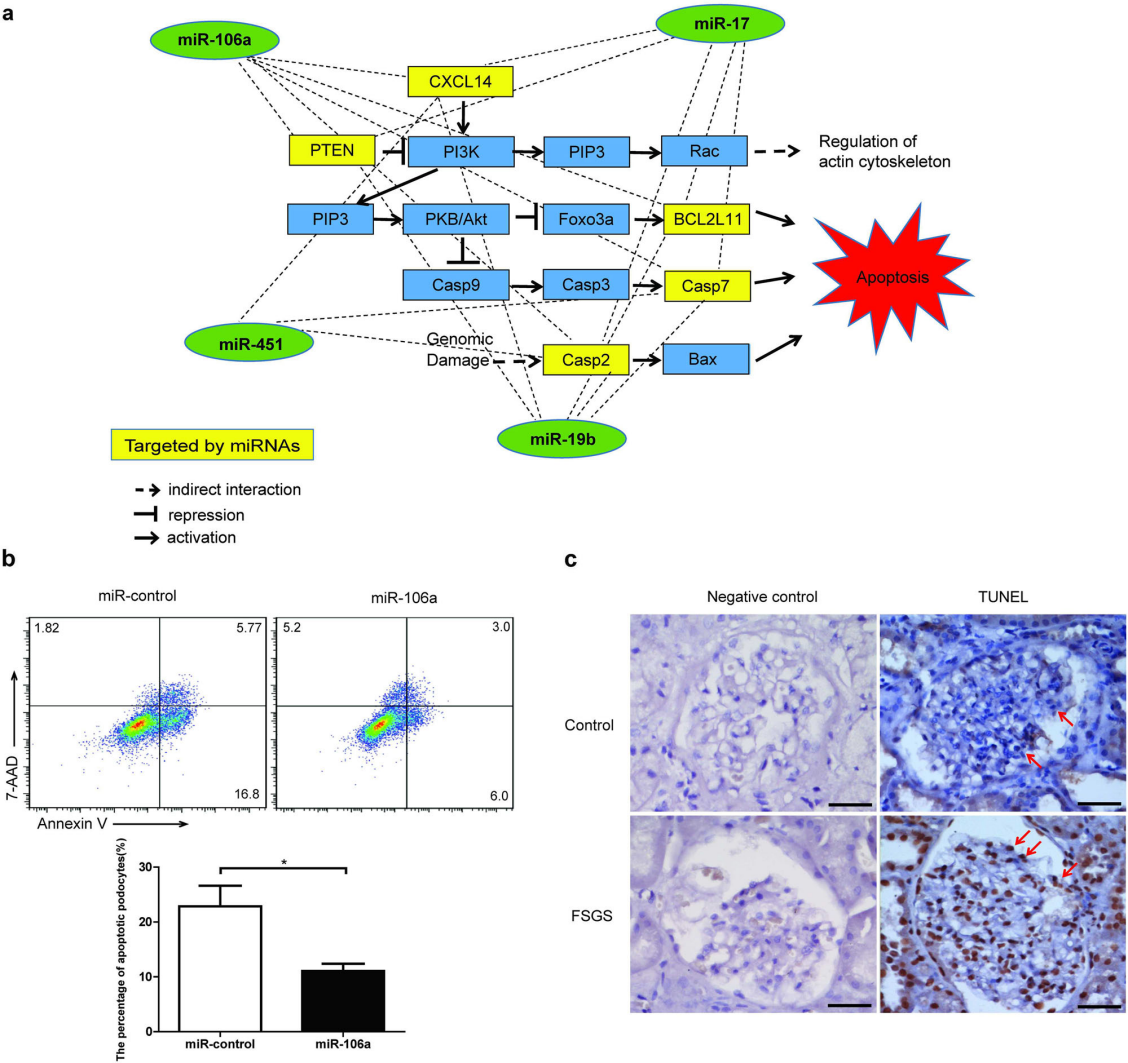
Overexpression of miR-106a suppresses the apoptosis of human podocytes in vitro. **a** Target genes and biological pathways for validated four-miRNA panel were identified using prediction algorithm (MICRORNA.ORG) and KEGG pathway enrichment. **b** Human podocyte cells were transfected with miR-106a or miR-control. The apoptotic rates of these cells were assessed by annexin V/7AAD staining. One representative flow cytometry analysis is shown. The bar graph shows the mean ± SD of three independent experiments. **c** Apoptosis in situ in FSGS renal biopsies and normal renal tissues were measured by TUNEL. Podocytes are marked by an arrowhead. Scale bars = 20 μm. *P *<* 0.05

The above pathway analysis suggested apoptosis signaling pathway was associated with four-miRNA panel, and some identified target genes, including PTEN and BCL2L11, were involved in the regulation of apoptosis pathway (Fig. 8a). So we tried to investigate the role of miRNAs in apoptosis of podocyte. Firstly, we confirmed the expression of miR-106a in FSGS patients via in situ hybridization (ISH). We found that miR-106a was not only expressed in podocytes, but also in other cell types, including endothelial cells, mesangial cells, and kidney tubules. Consistent with RT-PCR results, ISH results showed miR-106a was significantly downregulated in FSGS tissues compared with normal renal tissues (Figure S5). Then we transiently transfected miR-106a mimics or miRNA control into human podocyte cell line. The efficacy of miR-106a overexpression was confirmed by RT-PCR (Figure S6). Flow cytometry results showed that miR-106a overexpression led to reduced apoptosis rate in podocytes (Fig. 8b). The dysfunction of podocyte has emerged as a central mechanism underlying FSGS injury and sclerosis. We tested whether increased apoptosis in podocyte can be observed by using TdT-mediated dUTP nick-end Labeling (TUNEL) assay. FFPE tissues from FSGS patients exhibited significant increase in TUNEL-positive cells compared with normal kidney tissues (Fig. 8c). Above data suggest that miR-106a can suppress podocyte apoptosis in vitro.

## Discussion

Recently, a wealth of publications have demonstrated that circulating miRNAs may serve as novel biomarkers in various diseases. Here, we found four-plasma miRNAs (miR-17, miR-451, miR-106a, and miR-19b) were significantly downregulated in FSGS compared with healthy controls. The four-miRNA panel can produce an AUC value of 0.82. Moreover, plasma-miRNA panel was associated with FSGS severity and histologic classification. miR-17, miR-451, and miR-106a were related to FSGS remission. Taken together, our data show that the plasma-miRNA panel is a potential diagnostic and prognostic factor for FSGS. In recent study from Zhi-Hong Liu’s group, other plasma miRNAs such as miR-125b, miR-186, and miR-193a-3p were found to be upregulated in FSGS patients^17^. This suggests that FSGS is a heterogeneous disease and it needs us to use larger cohort to validate the biomarker.

An ideal biomarker is not only specific, and sensitive, but also can reflect the stage or prognosis of the disease. According to eGFR, FSGS is always divided into five stages and each stage has different degrees on kidney damage and different treatment. In current study, we found that the expression of plasma miR-17, miR-451, and miR-19b was significantly lower in medium-severe FSGS compared with mild FSGS. In addition, there was a correlation between miRNAs expression (especially for miR-17 and miR-19b) and histologic classification of FSGS. However, no correlation was found between miRNA and clinical parameters including creatinine, eGFR, proteinuria, serum albumin, and treatment type. Above data suggest that plasma-miRNA panel is an independent biomarker for FSGS, and is associated with FSGS progression.

For adult primary FSGS patients, steroid treatment is the first-line treatment. However, treatment responsiveness and recurrence remains a therapeutic challenge for the physician. In this study, we found the expression of the three-miRNA panel, including miR-17, miR-451, and miR-106a was associated with complete remission of FSGS.

Moreover, the expression of four-miRNA panel was significantly increased in FSGS remission group compared with untreated FSGS (data not shown). Now we are performing a prospective study to determine whether miRNA panel is the predictor of treatment response. Predicting remission or treatment responsiveness is essential for the early identification of high-risk individuals who need individualized treatment.

To determine whether the downregulated expression of four-miRNA panel was specific to FSGS, we enrolled IgAN, MSPGN, and MN patients as disease controls. Interestingly, we did not observe significant downregulation of four-miRNA panel in plasma samples from disease controls compared with health controls. To date, increasing evidence has shown that differently expressed circulating miRNAs can be found in various kidney diseases, for example, acute kidney injury^18^, kidney fibrosis^19^, idiopathic nephrotic syndrome^20^, diabetic nephropathy^13^, IgAN^14^, and lupus nephritis^15^. However, above reports did not show the reduced plasma expression of miR-17, miR-451, miR-106a, and miR-19b in disease groups, suggesting the downregulation of four-miRNA panel is specific for FSGS and may be involved in the pathogenesis of FSGS.

Now the molecular mechanism of FSGS remains unclear. Identification of four-miRNA panel may provide new insights into the pathogenesis of FSGS. According to available evidence, above miRNAs seem to be involved in kidney development, homeostasis, and disease. MiR-17 and miR-19b, as members of miR-17 ~ 92 cluster, play key roles in kidney development and homeostasis. Mice deficient in miR-17 ~ 92 will develop glomerular dysfunction and proteinuria^21^. MiR-451 has been demonstrated to inhibit glomerular mesangial cell proliferation by regulating p38 MAPK signaling in diabetic nephropathy^22^. One study in experimental animal model has found miR-17 and miR-106a were activated during the maintenance and recovery phases of renal ischemia-reperfusion injury^23^.

To explore the function of four-miRNA panel in FSGS, we performed targets prediction and pathway analysis. We interestingly found many genes involved in apoptosis signaling pathway such as PTEN, BCL2L11, and Casp7 may be co-regulated by four-miRNA panel. Consistent with our data, the identified four miRNAs have been reported to play roles in both the apoptotic and cell-proliferation pathways^24–26^. Podocyte loss is a hallmark of FSGS, and apoptosis is one of the major causes of podocyte loss. Now the mechanism underlying podocyte apoptosis remains incompletely understood. Among the identified four miRNAs, we selected miR-106a as a representative for further functional study. We found overexpression of miR-106a in human podocytes led to enhanced apoptosis. In addition, other groups’ data also suggest miRNA can regulate the apoptosis of podocyte, for instance, miR-30a was found to inhibit podocyte apoptosis^6^, while miR-195 was shown to promote podocyte apoptosis^27^.

PTEN, BCL2L11, and CXCL14 were identified as direct target genes of miR-106a in podocyte, suggesting miR-106a may regulate podocyte apoptosis by negatively regulating target genes. PTEN, as a well known tumor suppressor protein, can promote cell cycle arrest and apoptosis^28^. BCL2L11, commonly called BIM, belongs to the BCL-2 protein family and promotes apoptosis^29^. CXCL14 is one of the CXC C-X-C motif family and mainly contributes to the regulation of immune cell migration. Previous reports have found that elevated expression of CXCL14 in FSGS was able to attract inflammatory cells through the glomerulus, and it was another potential mechanism of inflammation and fibrosis^30,31^. In current study, we also found the mRNA and protein levels of PTEN, BCL2L11, and CXCL14 were upregulated in FSGS tissues compared with normal renal tissues.

Although, accumulating evidence indicates that circulating miRNAs may serve as important invasive biomarkers, the molecular mechanisms underlying the secretion of the plasma miRNAs remains elusive. miRNAs can be selectively packaged using exosomes, multivesicular bodies, or other RNA-binding proteins related pathways. Our hypothesis is that the reduced expression of four-miRNA panel is associated with the pathogenesis and outcome of FSGS. On the one hand, the downregulation of miRNAs such as miR-106a can result in the enhanced apoptosis of podocyte through targeting PTEN and BCL2L11, leading to the loss of podocyte and development of FSGS. On the other hand, above miRNAs can be involved in the inflammation and fibrosis processes during FSGS through targeting CXCL14.

In summary, we have identified circulating miRNA panel as a novel diagnostic and prognostic biomarker for FSGS. Above miRNAs are involved in FSGS disease pathogenesis through regulating podocyte apoptosis. A prospective large-scale study is under way to validate the potential utility of miRNAs biomarker. We hope the miRNA panel is helpful to early diagnosis, evaluation of treatment outcome, and personalized treatment for FSGS.

## Materials and methods

### Samples collection and experimental design

The study population consisted of 102 biopsy-confirmed FSGS patients and 129 healthy controls. A total of 69 IgAN patients, 24 MSPGN patients, and 26 MN patients were enrolled as disease controls. Recruited FSGS patients were idiopathic FSGS. Inclusion criteria were aged 16–70 years, free of individuals with secondary FSGS, IgAN, MSPGN, and MN. Individuals with severe disorders of the heart, brain, liver, or the hematopoietic system, pregnancy, lactation, cancer, and concurrence of other chronic diseases were excluded from the study. Healthy controls were volunteers who showed no abnormalities during the medical checkup in Southwest Hospital and had no known kidney disease history. In addition, FFPE samples from 20 FSGS and 20 normal kidney tissues were recruited for this study. Normal kidney tissues were obtained from histologically normal tissues flanking surgically excised renal tumors. All samples were collected from Southwest Hospital of Third Military Medical University (Chongqing, China) between 2013 and 2016. Written informed consent was obtained from all patients and the protocols were approved by the Ethics Review Board at Third Military Medical University. The discovery set consisted of five FSGS patients and five healthy controls. The training set consisted of 24 FSGS patients and 35 healthy controls. The validation set consisted of 50 FSGS patients, 68 healthy controls, and 119 disease controls. Additionally, 23 FSGS patients and 21 healthy controls were included in blinded test. Clinical characteristics of patients are given in Table S6.

### Cell culture

The human podocyte cell line was provided by Moin A Saleem (University of Bristol, Bristol, United Kingdom), and the cells were cultured as previously described^32^. The podocytes were treated with RPMI 1640 medium added with 10% fetal calf serum and 1% ITS Liquid Media Supplement (Sigma Chemicals), which contains 1.0 mg/ml recombinant human insulin, 0.55 mg/ml human transferrin (substantially iron-free), and 0.5 μg/ml sodium selenite at the 100× concentration. HEK293 cells were cultured in DMEM plus 10% FBS at 37 °C and 5% CO2.

### RNA extraction

For blood samples, plasma was isolated from EDTA-anticoagulated blood samples using a two-step centrifugation protocol (2000 r.p.m. for 10 min, 12,000 r.p.m. for 3 min). Total RNAs were extracted from 400 µl of plasma using the mirVana PARIS Kit (Applied Biosystems, Foster City, CA, USA) according to the manufacturer’s instructions. We used synthetic Caenorhabditis elegans miRNA(cel-miR-39) (RiboBio, Guangzhou, China) as a spiked-in normalization control. A volume of 10 µl of 50 nM cel-miR-39 was added to each denatured sample. For FFPE tissues, total RNA was extracted by PureLink FFPE Total RNA Isolation Kit (Life Technologies, Carlsbad, CA, USA) according to the manufacturer’s instructions. RNA quality was monitored by using Nanodrop 1000.

### Real-time qPCR-based high-throughput miRNA profiling

We used a real-time qPCR-based high-throughput miRNA profiling (QuantoBio, Beijing, China) that contains primers specific for 515 well-characterized human miRNAs to compare the plasma-miRNA profiles of five FSGS patients and five healthy controls. Cel-miR-39 was used for data normalization.

### qRT-PCR

For qRT-PCR of individual miRNA and mRNA, TaqMan probe-based RT-PCR was carried out according to previously described methods^33^ and manufacturer’s protocol. Taqman assays ID used in this study are shown in Table S7.

For qRT-PCR of individual miRNA, cDNA was synthesized in 5 µl volumes containing 1.67 µl of RNA, 0.5 µl of 10X reverse transcription buffer, 0.05 µl of 100 mM dNTPs, 0.063 µl of RNase inhibitor (20 U/µl), 0.33 µl of mutiscribe reverse transcriptase (50 U/µl), 0.5 µl of stem-loop primer, and 1.887 µl of nuclease-free water. Then cDNA was diluted 2 fold by nuclear-free water. Quantitative PCR reactions were carried out using 2 µl of cDNA solution, 5 µl of Premix Ex Tq^TM^ (TaKaRa Biotechnology, Dalian, China), 0.25 µl of gene-specific primers/probe (Applied Biosystems), and 2.75 µl of water in a final volume of 10 µl, and run on CFX96 thermocycler (Bio-Rad, Hercules, CA, USA). For plasma samples, cel-miR-39 was used for normalization. For FFPE tissues, U6 snRNA served as endogenous control. Relative expression was calculated using the 2^−⊿⊿*C*t^ method.

For qRT-PCR of mRNA, RNA was reverse transcribed using PrimeScript RT reagent Kits (TaKaRa). TaqMan probe-based qRT-PCR of BCL2L11, CXCL14, PTEN and 18sRNA were performed via TaqMan Gene Ex Assays (Applied Biosystems) and Premix Ex TqTM (Takara) according to the manufacturer’s protocol.

### TUNEL assay

TUNEL staining was carried out according to previously described methods^34^. FFPE sections were deparaffinized and rehydrated, and then were stained with hematoxylin and eosin. Apoptotic cells were stained by using an in situ cell death detection kit, POD (Roche Diagnostics, Shanghai, China). Negative control was subjected to the same staining for TUNEL without TdT. Images were collected by using microscope with ×400 magnification.

### Immunohistological analysis

Human kidney biopsy tissues were fixed in formalin and embedded in paraffin. The sections were deparaffinized, rehydrated, incubated in 3% H_2_O_2_ for 10 min in the dark at room temperature to block the endogenous peroxidase activity. Antigen retrieval was performed by heating in a microwave oven with citrate buffer (pH 6.0). Subsequently, the slides were blocked with 10% fetal bovine serum in PBS for 15 min at 37 °C, followed by incubation at 4 °C overnight with the primary antibodies (CXCL14 at 1:25 dilution; PTEN at 1:100 dilution; BCL2L11 at 1:50 dilution) (Abcam, Cambridge, MA, USA) overnight at 4 °C. Then biotinylated secondary antibody, Sav-HRP conjugates (Zhongshan Biotechnology, Beijing, China), and DAB substrate solution (Roche Diagnostics) were used according to the manufacturer’s protocol. Images were collected by using microscope with ×400 magnification.

### miRNA ISH

Digoxigenin (DIG)-labeled RNA probes for miR-106a-5p were purchased from Pengekiphen (Suzhou, China). Human renal biopsies slides were taken out of-40 °C freezer and warmed up for 15 min at 50 °C incubator. Human renal biopsies slides were incubated with DIG-labeled RNA probes in a humid box for overnight at 41 °C, miR-16-5p as a positive control. After washing, slides were incubated with anti-DIG antibody conjugated with alkaline phosphatase (1:2000) in blocking buffer for overnight at 4 °C and followed by color development with NBT/BCIP (Roche) as substrates.

### Western blot assay

Samples were separated by 10% SDS-polyacrylamide gel electrophoresis and transferred to polyvinylidene fluoride membranes. BCL2L11 and PTEN proteins were detected with anti-BCL2L11 and anti-PTEN rabbit monoclonal Abs (1: 1000; Abcam). CXCL14 proteins were detected with anti-CXCL14 rabbit polyclonal Abs (1:1000; Abcam). Mouse monoclonal GAPDH antibody (1: 2000; Cell Signaling Technology, Beverly, MA, USA) was used as internal reference. HRP-conjugated secondary antibody (1:10000) was purchased from Zhongshan Biotechnology. Bound proteins were visualized by using SuperSignal West Dura Extended Duration Substrate kit (Thermo Scientific, Wilmington, DE, USA).

### Cell apoptosis assay

The percentage of apoptotic human podocytes was assessed by using flow cytometery. The conditionally immortalized human podocytes were seeded in six-well plate at the density of 3.5 × 10^5^ cells/well and grown to 70 or 80 % confluency in 33 °C, and then were cultured in 37 °C to differentiate. Human podocytes were transfected with miR-106a mimics or miR-control (RIBOBIO) at a concentration of 50 nM. After 24 h, cells were collected and double-stained by annexin V/7AAD (BioLegend, San Diego, CA, USA), and the percentage of apoptotic human podocytes was assessed. The results were analyzed by FlowJo software version 6.2.

### Luciferase assay

The construction of luciferase report vectors was performed as previously described^35^. The complementary 75-mer DNA oligonucleotides of CXCL14 3′-UTR sequence containing the putative miR-106a target sites were synthesized with flanking *Spe* I and *Hind* III restriction enzyme digestion sites (sense: 5′-CTAGTGGGCCCGACACAAATTATATATTGTTATGAAGCACTTTTTACCAACGGTCAGTTTTTACATTTTATAGCA-3′; antisense: 5′-AGCTTGCTATAAAATGTAAAAACTGACCGTTGGTAAAAAGTGCTTCATAACAATATATAATTTGTGTCGGGCCCA-3′). The mutant seed regions were generated as: GCACTTT to TAATCGT. HEK293T cells were seeded onto 96-well plates (1 × 10^4^ cells per well) the day before transfection. Cells were co-transfected with the wild-type or mutant luciferase reporter vector (200 ng/well), and pRL-TK (10 ng/well) (Promega, Madison, WI, USA), and miR-106a mimics or miR-control (50 nM). Cell lysates were prepared with Passive Lysis Buffer (Promega) 48 h after transfection, and luciferase activities were measured by using the Dual Luciferase Reporter Assay (Promega). The firefly luciferase activity was normalized to the renilla luciferase activity.

### Statistical analysis

The Mann–Whitney test was used to compare the differences in plasma-miRNA expression between FSGS and control groups. ROC curves were used to assess the sensitivity and specificity of miRNA biomarkers. Logistic regression was used to develop a combined miRNA panel as previously described^36^. Cox regression was performed to evaluate the association between miRNAs and FSGS remission. Spearman correlation analysis was performed to examine the relationship between four-miRNA panel and clinical parameters. Colinearity diagnostics analysis was performed to evaluate the influence of correlation within four miRNAs on the diagnostic model. DAVID analysis was used to search the common pathways shared with the four-miRNA biomarker panel. All statistical analysis was performed using SPSS 16.0 software, and graphs were generated using GraphPad Prism 5.0.

## Electronic supplementary material

Supplementary material
