# Supplementary material for: Plasma microRNA panel is a novel biomarker for focal segmental glomerulosclerosis and associated with podocyte apoptosis
Source: Cell Death Dis. 2018 May 10;9(5):533. doi: 10.1038/s41419-018-0569-y (PMC5945632; doi:10.1038/s41419-018-0569-y)
Supplement: Supplementary file 1 — Supplementary material [file 41419_2018_569_MOESM1_ESM.pdf]

Supplemental Tables

Table S1. The delta Ct values of differentially expressed miRNAs in the screening phase

	F1	F2	F3	F4	F5	N1	N2	N3	N4	N5
**miR-508-5p**	7.11	9.19	6.20	8.48	7.23	10.90	9.42	12.18	10.93	9.54
**miR-19a-5p**	9.70	9.74	11.21	3.94	10.28	10.97	12.69	10.97	11.59	12.07
**miR-106b-3p**	6.66	8.04	7.21	10.06	8.98	10.04	9.91	11.06	10.44	10.58
**miR-323-3p**	10.74	10.86	11.50	11.01	12.58	13.80	12.41	12.96	12.40	16.16
**miR-499-5p**	9.46	11.87	8.11	11.82	13.21	13.24	13.15	12.77	13.27	13.03
**miR-139-3p**	6.33	6.75	6.04	8.36	9.30	7.45	10.80	9.37	10.51	8.93
**miR-338-5p**	9.50	11.48	11.00	11.13	13.77	13.23	13.74	14.66	13.95	11.20
**miR-877-5p**	8.40	10.25	10.00	8.82	10.02	10.98	11.61	11.95	11.26	11.38
**miR-1233-3p**	8.41	5.58	9.32	6.92	5.70	9.48	8.18	8.34	10.04	9.14
**miR-720**	3.25	5.48	3.78	2.30	4.46	5.97	5.53	7.94	4.66	4.22
**miR-1469**	11.39	13.52	11.37	11.86	12.57	14.48	13.11	14.40	13.92	13.70
**miR-381-3P**	5.66	7.29	6.81	7.72	8.68	8.31	10.02	8.49	9.16	8.06
**miR-301a-5p**	11.04	9.06	8.65	11.00	11.42	12.67	11.61	11.56	11.60	11.51
**miR-323-5p**	9.07	11.64	10.17	9.98	11.22	12.45	11.89	11.89	11.62	11.57
**miR-1229-5p**	10.88	12.40	10.87	10.40	11.64	13.68	11.45	12.80	12.55	12.81
**miR-572**	6.97	7.68	6.61	6.58	7.50	8.37	8.18	8.58	8.34	7.56
**miR-324-5p**	11.05	12.57	11.65	11.35	12.10	11.20	10.62	11.03	10.30	9.69
**miR-15b-5p**	7.29	9.03	8.33	7.98	8.33	7.80	7.11	7.33	6.56	6.22
**miR-532-5p**	10.44	11.48	11.75	11.04	11.88	11.01	10.26	10.41	9.76	8.79
**miR-500**	11.59	12.82	11.97	11.62	12.44	11.87	10.77	11.00	10.41	9.82
**miR-423-5p**	9.93	11.11	10.69	9.55	10.62	9.92	9.40	9.64	8.50	7.82
**let-7i-5p**	9.09	10.05	9.55	9.86	9.92	9.33	8.34	7.96	8.14	7.16
**miR-106b-5p**	8.13	8.89	9.36	9.96	8.95	8.48	7.22	7.95	7.62	6.14
**miR-106a-5p**	6.10	6.77	7.56	7.46	6.80	6.62	4.63	5.66	5.08	4.68
**miR-103**	8.58	10.23	10.49	10.05	10.37	8.58	7.70	8.89	8.20	8.10
**miR-598**	10.16	11.12	12.33	12.59	11.00	10.63	9.47	10.07	9.08	9.45
**miR-185-5p**	6.96	8.42	8.06	7.98	7.93	7.13	6.27	6.61	5.85	4.89
**miR-19b-3p**	4.43	5.50	5.81	5.34	5.37	4.91	3.60	3.94	2.85	2.29
**miR-17-5P**	8.55	9.50	10.36	10.67	9.74	9.02	7.46	8.13	7.51	7.67
**miR-18a-5p**	9.19	9.94	10.57	10.61	10.10	9.38	7.91	8.64	8.01	7.15
**miR-451a**	-0.82	-0.02	0.98	0.96	0.06	-0.36	-1.52	-1.77	-2.46	-2.26
**miR-363-3p**	8.43	9.05	8.71	8.72	9.52	8.53	7.05	6.99	6.51	5.65
**miR-18b-5p**	11.73	12.58	12.74	12.59	12.61	11.48	10.29	11.20	10.04	9.27
**miR-210**	20.82	11.57	12.18	11.39	11.59	10.96	10.44	10.37	9.62	9.02

Table S2. Kruskal-Wallis analysis between the levels of 4 miRNAs and different histologic subtypes of FSGS

	**miR-17**	**miR-19b**	**miR-106a**	**miR-451**
***P* value (K-W *H* test)**	0.043	0.011	0.072	0.123

Table S3. Cox regression analysis for the estimation of association between plasma miRNA panel and the remission of FSGS

	Univariate analysis			Multivariate analysis		
	OR	95% CI	*P*	OR	95% CI	*P*
Age	0.988	0.953-1.025	0.516			
Sex (Male/Female)	1.463	0.495-4.321	0.491			
Treatment type	0.176	0.021-1.449	0.106			
Serum albumin	1.110	1.017-1.211	0.019	1.113	1.013-1.224	0.025
Creatinine	0.980	0.965-0.995	0.009	0.951	0.903-1.001	0.055
eGFR	1.016	1.002-1.030	0.026	0.959	0.901-1.021	0.191
CKD score	0.419	0.219-0.800	0.008	0.597	0.048-7.381	0.688
miRNA panel	2.990	1.113-8.036	0.030	2.868	1.090-7.545	0.033

Table S4. Correlation analysis for miRNA panel by SPSS 16.0

		miR-17	miR-451	miR-106a	miR-19b
**miR-17**	**Correlation Coefficient**	1.000	0.773	0.880	0.898
	**Sig. (2-tailed)**	\	< 0.001	< 0.001	< 0.001
**miR-451**	**Correlation Coefficient**	0.773	1.000	0.727	0.722
	**Sig. (2-tailed)**	< 0.001	\	< 0.001	< 0.001
**miR-106a**	**Correlation Coefficient**	0.880	0.727	1.000	0.843
	**Sig. (2-tailed)**	< 0.001	< 0.001	\	< 0.001
**miR-19b**	**Correlation Coefficient**	0.898	0.722	0.843	1.000
	**Sig. (2-tailed)**	< 0.001	< 0.001	< 0.001	\

Table S5. Correlation analysis between 4-miRNA panel and clinical parameters by SPSS 16.0

		Creatinine	eGFR	Proteinuria	Serum albumin	Treatment type
4-miRNA panel	Correlation Coefficient	0.154	-0.165	0.029	-0.109	-0.010
	Sig. (2-tailed)	0.191	0.159	0.804	0.356	0.931

Table S6. The common pathways shared with the 4-miRNA biomarker panel

KEGG pathway	P value	No. of genes	No. of miRNA
Pathway in cancer,hsa05200	8.8E-4	22	4
PI3K-Akt signaling pathway,hsa04151	4.2E-4	21	4
FoxO signaling pathway,hsa04068	1.4E-4	13	4
Focal adhesion,has04510	3.6E-2	11	4
Calcium signaling pathway,hsa04020	1.5E-2	11	4

Table S7. Clinical characteristics of patients recruited in the study

	FSGS (n=102)	Healthy control (n=129)	Disease controls		
			IgAN (n=69)	MSPGN (n=24)	MN (n=26)
Median Age (years)	35.0	33.0	35.0	39.5	49.0
Gender (% female)	56.9	46.5	55.1	41.7	60.0
Creatinine (μmol/l)	115.8	79.0	98.4	83.8	78.5
eGFR (ml/min per 1.73 m^2)	78.3	113.2	86.9	101.9	89.6

Table S8. Taqman assays used in this study

Gene	Assay ID
miR-17	002308 (Taqman miRNA assay)
	478447 (Taqman advanced miRNA assay)
miR-451	001141 (Taqman miRNA assay)
	478107 (Taqman advanced miRNA assay)
miR-106a	002169 (Taqman miRNA assay)
	478225 (Taqman advanced miRNA assay)
miR-19b	000396 (Taqman miRNA assay)
	478264 (Taqman advanced miRNA assay)
cel-39	000200 (Taqman miRNA assay)
	478293 (Taqman advanced miRNA assay)
PTEN	Hs02621230_s1
BCL2L11	Hs00708019_s1
CXCL14	Hs01557413_m1
CASP7	Hs00169152_m1
COL1A2	Hs01028956_m1
18sRNA	Hs03003631_g1

Supplemental Figures

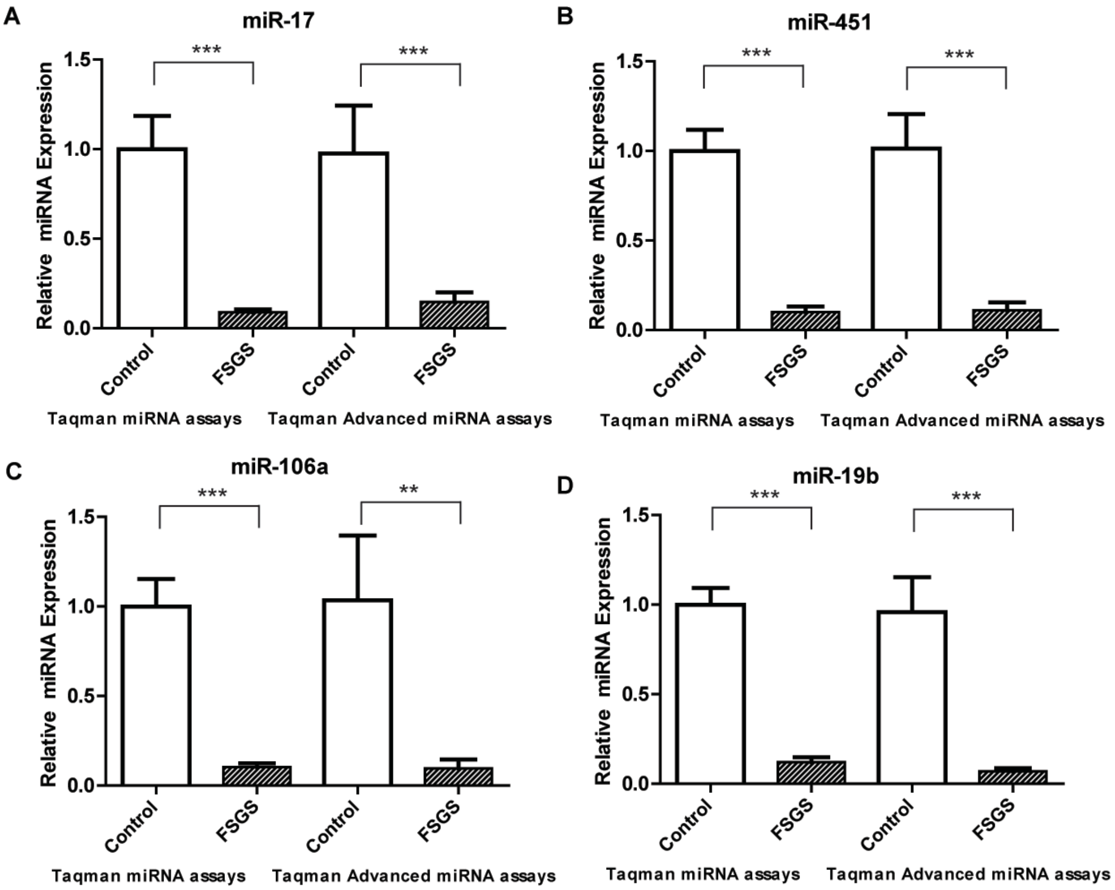

Figure S1. Comparison of 4-miRNA panel expression by using two different Taqman qPCR systems. We analyzed the expression of 4-miRNA panel in 20 FSGS plasmas and in 20 healthy controls by using Taqman advanced miRNA assay. **, $P < 0.01$. ***, $P < 0.001$.

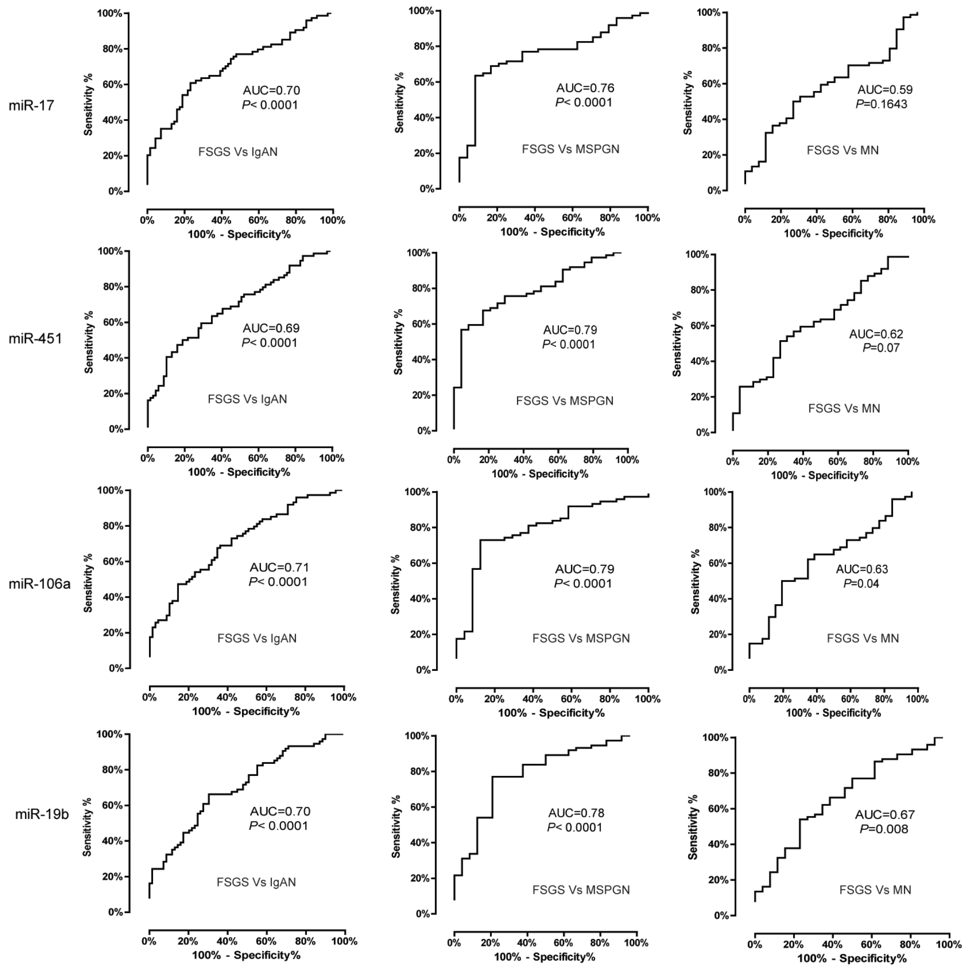

Figure S2. ROC analysis of 4-miRNA panel for the discrimination between FSGS (n=74) and disease controls including IgAN (n=69), MSPGN (n=24), and MN (n=26).

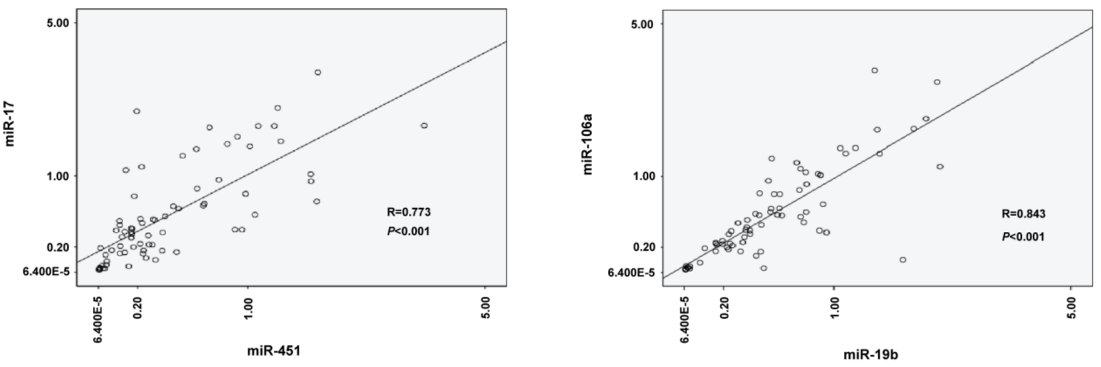

Figure S3. Correlation analysis of miR-17, miR-451, miR-106a and miR-19b. Significant positive correlations were found between miR-17 and miR-451, and between miR-106a and miR-19b in plasma from FSGS patients (n=74).

Coefficients[a]

Model		Unstandardized Coefficients B	Unstandardized Coefficients Std. Error	Standardized Coefficients Beta	t	Sig.	Collinearity Statistics Tolerance	Collinearity Statistics VIF
1	(Constant)	-.515	.292		-1.764	.080		
	miR-17	-.130	.056	-.433	-2.340	.020	.135	7.401
	miR-451	.064	.026	.264	2.416	.017	.388	2.575
	miR-106a	.047	.055	.154	.842	.401	.138	7.236
	miR-19b	.125	.039	.441	3.183	.002	.241	4.157

a. Dependent Variable: Disease

Collinearity Diagnostics[a]

Model	Dimension	Eigenvalue	Condition Index	Variance Proportions (Constant)	miR-17	miR-451	miR-106a	miR-19b
1	1	4.958	1.000	.00	.00	.00	.00	.00
	2	.030	12.903	.16	.00	.47	.00	.00
	3	.008	25.486	.72	.02	.50	.03	.18
	4	.003	37.918	.13	.16	.00	.11	.80
	5	.001	62.423	.00	.82	.03	.86	.02

a. Dependent Variable: Disease

Figure S4. The multicollinearity analysis of 4-miRNA panel by SPSS.

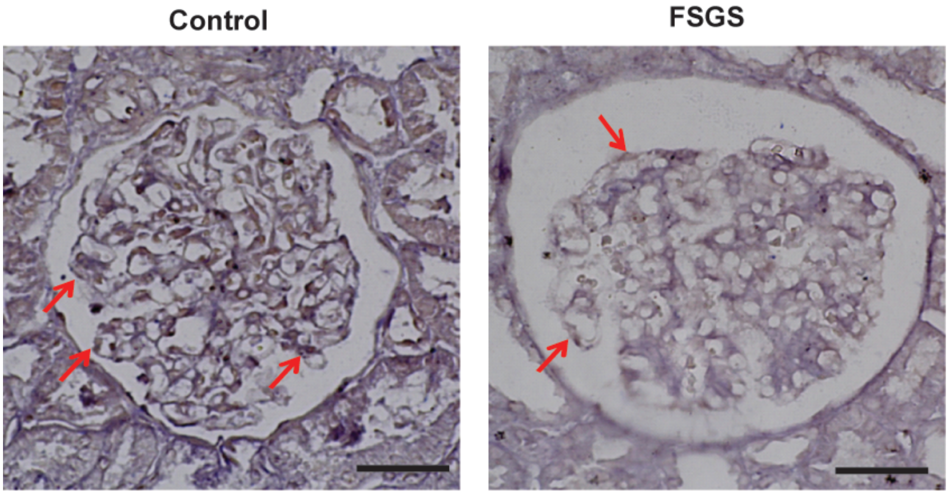

Figure S5. **In situ hybridization of miR-106a in FSGS renal biopsies and normal renal tissues.** Podocytes are marked by an arrowhead. Scale Bars = 20 μm.

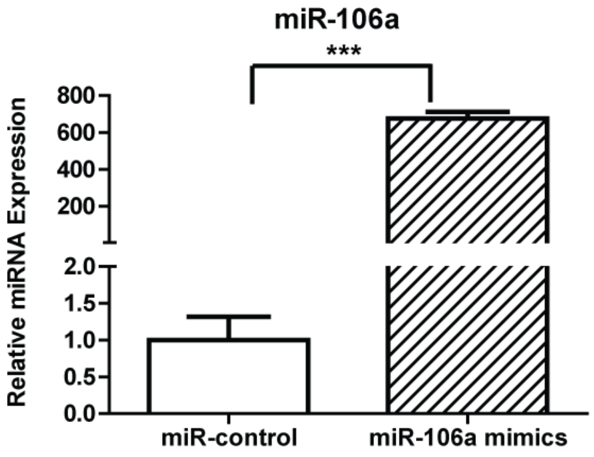

Figure S6. **The efficacy of miR-106a overexpression in human podocytes.** The human podocyte cell line was transfected with miR-106a mimics or miR-control (50 nM). The efficacy of miR-106a overexpression was confirmed by qRT-PCR. Data are presented as mean±S.D. (n=3). ***, $P < 0.001$.
